# Tumor location may affect the clinicopathological features and prognosis of thymomas

**DOI:** 10.1111/1759-7714.13188

**Published:** 2019-09-09

**Authors:** Dong Tian, Haruhiko Shiiya, Masaaki Sato, Chang‐Bo Sun, Masaki Anraku, Jun Nakajima

**Affiliations:** ^1^ Department of Thoracic Surgery The University of Tokyo Graduate School of Medicine Tokyo Japan; ^2^ Department of Thoracic Surgery Affiliated Hospital of North Sichuan Medical College Nanchong China; ^3^ Department of Thoracic Surgery, West China Hospital Sichuan University Chengdu China; ^4^ Department of Cardiovascular and Thoracic Surgery Hokkaido University Graduate School of Medicine Sapporo Japan

**Keywords:** Clinicopathological features, prognosis, recurrence, thymoma, tumor location

## Abstract

**Background:**

The current staging systems do not consider the tumor location of thymomas, and its clinical relevance is poorly understood. This study aimed to evaluate the impact of tumor location on the clinicopathological features and prognosis of thymomas.

**Methods:**

We performed a retrospective review of patients at our institution who underwent an extended thymectomy for a thymoma from 1976 to 2015. The tumor location was classified as either the superior or inferior mediastinum based on the maximum tumor diameter. The clinicopathological characteristics of the thymoma were also evaluated. Kaplan‐Meier estimates and Cox proportional hazards models were used to analyze the survival outcomes and risk factors for recurrence.

**Results:**

A total of 194 patients with thymoma were eligible for this study. Compared with the inferior mediastinum group (*n* = 167), the superior mediastinum group (*n* = 27) had a higher frequency of myasthenia gravis (MG), advanced Masaoka‐Koga staging, disease progression and recurrence (*P* < 0.05). The Kaplan‐Meier analysis demonstrated thymomas in the superior mediastinum had worse survival outcomes that included overall survival, progression‐free survival and disease‐free survival (*P* < 0.05). The multivariate analysis showed tumor location was an independent prognostic factor for all the survival outcomes (*P* < 0.05). Furthermore, the tumor location (*P* = 0.004) and Masaoka‐Koga stage (*P* < 0.001) were the only two independent risk factors for recurrence in the multivariate analysis.

**Conclusions:**

The clinicopathological features of thymomas on MG, Masaoka‐Koga staging, disease progression, and recurrence were different between locations of superior and inferior mediastinum locations. Thymomas in the superior mediastinum tended to be associated with worse survival and increased recurrence.

## Introduction

Thymomas are regarded as rare, low‐grade malignant tumors of the thymic epithelium that do not have a consensus treatment.^1^, ^2^ Approximately 1.3–2.2/10^6^ people are affected by thymomas, according to worldwide cancer statistics.^3^, ^4^, ^5^ However, up to half of the anterior mediastinal tumors are diagnosed as thymomas, which are widely considered to be the most common tumors in the anterior mediastinum.^6^

The typical clinical manifestations of thymomas are a mass that is identified on chest images with or without symptoms such as myasthenia gravis (MG), intermittent coughing, different degrees of chest pain, and vena cava syndrome.^7^ Although thymomas are considered to be malignant, their 10‐year survival rates are more than 90%.^8^, ^9^

The clinicopathological and prognostic risk factors for thymomas are difficult to confirm because of the heterogeneous nature of the disease.^10^ In fact, many retrospective case series have described its characteristics and assessed different prognostic factors for long‐term survival as well as risk factors for tumor recurrence. While the Masaoka‐Koga staging system, which is based on the extent of tumor invasion, implantation, lymph node and/or hematogenous metastases, is widely accepted as the most important independent prognostic and risk factor for recurrence for thymomas, there is no consensus on the roles of other factors.^9^, ^10^, ^11^, ^12^, ^13^, ^14^, ^15^ The International Association for the Study of Lung Cancer (IASLC) Staging Prognostic Factors Committee and International Thymic Malignancy Interest Group (ITMIG) recently proposed a staging system for thymomas and thymic carcinomas (TCs) that was in accordance with their survival outcomes.^16^ This staging system was approved for the eighth edition of the tumor, nodes, and metastasis (TNM) staging classification by the American Joint Committee on Cancer (AJCC) / the Union for International Cancer Control (UICC) consortium.^16^, ^17^, ^18^ Despite their extensive use, both the Masaoka‐Koga and the TNM staging systems have ambiguities that have not been confirmed. Although thymomas can occur in any location of the anterior mediastinum, differences based on their anatomical tumor location have not been described in either of the current staging systems. As a result, it is currently unknown if tumor location can impact the clinicopathological features and survival outcomes of this disease. This retrospective study aimed to describe the clinicopathological features of thymomas that were located in different tumor locations. We also analyzed patient clinical outcomes to determine if tumor location is an independent prognostic factor and a risk factor for recurrence.

## Methods

### Patients

The Ethics Committees and Review Board of the University of Tokyo Hospital approved this study (No. 2406), and the need for patient consent was waived. This retrospective review included all the patients at our institute with a thymoma who underwent an extended thymectomy from 1 January 1976 to 31 December 2015. The clinical information was retrieved from electronic (2000–2015) and archived paper charts (1976–1999). The inclusion criteria for patients in this study were as follows: (i) a pathologically confirmed thymoma; (ii) complete clinical and pathological data accessible for restaging based on the Masaoka‐Koga stage^19^; and (iii) the patient had undergone an extended thymectomy via a median sternotomy, as described in our previous studies.^20^ We excluded thymic carcinomas or neuroendocrine tumors because they are histopathologically and clinically distinct from thymomas.^21^, ^22^ During the 40‐year study period, 201 consecutive patients with thymomas were reviewed. Three patients with ambiguous WHO classifications^23^ and four patients that lacked detailed imaging of the tumor location were excluded from the cohort. The clinicopathological features and survival outcomes of 194 patients were included in this retrospective review. The evaluated variables included tumor location, age, tumor diameter, sex, MG, Myasthenia Gravis Foundation of America (MGFA) classification, anti‐acetylcholine receptor (anti‐AchR), WHO classification, Masaoka‐Koga stage, multiple primary malignant tumors (MPMT), surgical radicality, lymph node dissection, lymph node metastasis, preoperative induction therapy, postoperative adjuvant therapy, disease progression, recurrence, overall survival (OS), progression‐free survival (PFS) and disease‐free survival (DFS).

### Definitions

The boundaries of the superior mediastinum were defined by previous publications as the level between the sternal angle to the inferior margin of the fourth thoracic vertebrae.^24^, ^25^ The tumor location was determined from preoperative thoracic imaging examinations and were based on the maximum diameter of the tumor from electronic images (year 2000–2015) or from the descriptions on paper images (year 1976–1999). The tumor diameter of each thymoma was measured by pathologists who recorded its value in the clinical charts. The presence of MG or MPMT was determined using preoperative information or data from the postoperative follow‐up period. Both the pre‐ and postoperative incidence of other cancers were included in this study. The MGFA classification^26^ was only performed for patients who experienced comorbid MG. The anti‐AchR was determined by preoperative radioimmunoassays. A classification of positive (more than 0.3 nmol/L) or negative (0.3 nmol/L or less) of anti‐AchR was used in accordance with the terms of the previous study.^27^ The staging and histological classification of thymomas were performed according to the Masaoka‐Koga staging system^19^ and the WHO histologic classification criteria.^23^ Surgical radicality was defined as either an R0 resection (with no residual tumor on microscopy) or a non‐R0 resection (with microscopic or macroscopic residual tumor). The preoperative induction therapy and postoperative adjuvant therapy consisted of either chemotherapy or radiotherapy. The definitions of disease progression, recurrence, OS, PFS and DFS were compliant with the standard outcome measures for thymic malignancies of the ITMIG.^28^ The disease progression, OS and PFS were analyzed for the whole population, while the recurrence and DFS were only calculated for patients who underwent an R0 resection.^28^

### Follow‐up

The postoperative follow‐up interval was every 3–6 months, and chest X‐ray or computed tomography (CT) were performed annually. Any other necessary examinations were determined by the patient's clinical needs. The final follow‐up visit was in October 2018 to evaluate survival and recurrence outcomes.

### Statistical analysis

Demographic and clinicopathologic variables were collected and included in the statistical analysis. The descriptive statistics are reported as the mean ± standard deviation. The categorical variables are reported as frequencies and proportions. The differences between the groups of superior and inferior mediastinum were assessed using a Student's *t*‐test, chi‐square test, linear‐by‐linear association, likelihood ratio detection or Fisher's exact test. The OS and PFS were evaluated using the Kaplan‐Meier method. The differences between survival curves were analyzed by a log‐rank test. Furthermore, patients who only underwent an R0 surgery were selected to evaluate DFS with the same methods described above. To assess the significance of tumor location as an independent prognostic risk factor for OS, PFS and recurrence, univariate and multivariate analysis were performed using the Cox's proportional hazards model. The hazard ratio (HR) was estimated with a 95% confidence interval (CI) for both the univariate and multivariate analysis. Since only a subset of the patients were comorbid for MG and underwent a lymph node dissection, we did not include the MGFA classification or lymph node metastasis as covariates in the univariate or multivariate analysis. The following variables were finally included as the starting set of covariates for the univariate analysis: tumor location (Superior/Inferior), age (as continuous), tumor diameter (as continuous), sex (male/female), MG (Yes/No), anti‐AchR (positive/ negative), WHO histologic classification (A/AB/B1/B2/B3), Masaoka‐Koga stage (I/II/III/IV), MPMT (Yes/No), surgical radically (R0 resection/non‐R0 resection), lymph node dissection (Yes/No), preoperative induction therapy (Yes/No), and postoperative adjuvant therapy (Yes/No). Only variables with a *P* < 0.1 in the univariate analysis were included as covariates for the multivariate analysis. Two‐sided tests were applied and a *P* < 0.05 was considered to be a statistically significant difference. The statistical analysis was performed using SPSS 24.0 (SPSS Inc., Chicago, IL, USA).

## Results

### Clinicopathological features

A total of 194 patients with thymomas were eligible to be included in this study, including 98 males and 96 females with a mean age of 53.2 ± 13.5 years versus 53.9 ± 14.7 years (*P* = 0.811), respectively. Patients with thymomas in the superior mediastinum group (*n* = 27) accounted for 13.9% of the total cases while those with thymomas in the inferior mediastinum group (*n* = 167) accounted for 86.1%. The clinicopathological characteristics of patients with superior and inferior mediastinum thymomas are shown in Table 1. There were no significant differences in age, tumor diameter, sex, MGFA classification, anti‐AchR, WHO histological classification, MPMT, surgical radicality, lymph node dissection, lymph node metastasis, preoperative induction therapy or postoperative adjuvant therapy between the two groups (*P* = 0.811, 0.448, 0.881, 0.492, 0.134, 0.069, 0.382, 0.721, 0.881, 0.268, 0.114, and 0.998, respectively). However, MG, Masaoka‐Koga stage, disease progression and recurrence were significantly different between the two groups (*P* = 0.007, 0.005, <0.001 and < 0.001, respectively). More patients with thymomas in the superior mediastinum had MG (55.6% vs. 29.3%), Masaoka‐Koga stage III/IV disease (40.7% vs.18.6%), and disease progression (44.4% vs. 8.4%) than those with thymomas in the inferior mediastinum. Only patients who underwent an R0 resection were included to evaluate recurrence. Patients with thymomas in the superior mediastinum tended to have a higher frequency of recurrence (37.5% *versus* 7.2%) than those with thymomas in the inferior mediastinum (*P* < 0.001).

**Table 1:** Summary of patient clinicopathologic characteristics in thymoma

Parameters	All patients (*n* = 194)	Superior (*n* = 27)	Inferior (*n* = 167)	*P‐*value
Age (years) (mean ± SD) (range)	53.8 ± 14.5 (15–83)	53.2 ± 13.5 (28–78)	53.9 ± 14.7 (15–83)	0.811^a^
Tumor diameter (cm) (mean ± SD) (rang)	5.2 ± 2.5 (1.0–19.4)	5.6 ± 3.1 (2.5–16.0)	5.2 ± 2.4 (1.0–19.4)	0.448^a^
Sex				0.881^b^
Male	98 (50.5%)	14 (51.9%)	84 (50.3%)	
Female	96 (49.5%)	13 (48.1%)	83 (49.7%)	
MG				0.007* ^,^ b
Yes	64 (33.0%)	15 (55.6%)	49 (29.3%)	
No	130 (67.0%)	12 (44.4%)	118 (70.7%)	
MGFA classification†,‡‡				0.492^c^
I	28 (14.4%)	8 (29.6%)	20 (12.0%)	
II	22 (11.3%)	4 (14.8%)	18 (10.8%)	
III	5 (2.6%)	1 (3.7%)	4 (2.4%)	
IV	5 (2.6%)	2 (7.4%)	3 (1.8%)	
V	4 (2.1%)	0 (0)	4 (2.4%)	
Anti‐AchR¶				0.134^b^
Positive	62 (32.0%)	12 (44.4%)	50 (29.9%)	
Negative	132 (68.0%)	15 (55.6%)	117 (70.1%)	
WHO classification‡				0.069^d^
A	17 (8.8%)	2 (7.4%)	15 (9.0%)	
AB	45 (23.2%)	4 (14.8%)	41 (24.6%)	
B1	64 (33.0%)	9 (33.3%)	55 (32.9%)	
B2	50 (25.8%)	5 (18.5%)	45 (26.9%)	
B3	18 (9.3%)	7 (25.9%)	11 (6.6%)	
Masaoka‐Koga stage§				0.005* ^,^ c
I	98 (50.5%)	9 (33.3%)	89 (53.3%)	
II	54 (27.8%)	7 (25.9%)	47 (28.1%)	
III	24 (12.4%)	5 (18.5%)	19 (11.4%)	
IV	18 (9.3%)	6 (22.2%)	12 (7.2%)	
MPMT				0.382^e^
Yes	27 (13.9%)	2 (7.4%)	25 (15.0%)	
No	167 (86.1%)	25 (92.6%)	142 (85.0%)	
Surgical radicality††				0.721^e^
R0 resection	176 (90.7%)	24 (88.9%)	152 (91.0%)	
Non‐R0 resection	18 (9.3%)	3 (11.1%)	15 (9.0%)	
Lymph node dissection				0.881^b^
Yes	41 (21.1%)	6 (22.2%)	35 (21.0%)	
No	153 (78.9%)	21 (77.8%)	132 (79.0%)	
Lymph node metastasis§§				0.268^e^
Yes	7 (3.6%)	2 (33.3%)	5 (14.3%)	
No	34 (96.4%)	4 (66.7%)	30 (85.7%)	
Preoperative induction therapy				0.114^e^
Yes	9 (4.6%)	3 (11.1%)	6 (3.6%)	
No	185 (95.4%)	24 (88.9%)	161 (96.4%)	
Postoperative adjuvant therapy				0.998^b^
Yes	79 (14.3%)	11 (40.7%)	68 (40.7%)	
No	115 (85.7%)	16 (59.3%)	99 (59.3%)	
Disease progression				<0.001* ^,^ b
Yes	26 (13.4%)	12 (44.4%)	14 (8.4%)	
No	168 (86.6%)	15 (55.6%)	153 (91.6%)	
Recurrence¶¶				<0.001* ^,^ b
Yes	20 (11.4%)	9 (37.5%)	11 (7.2%)	
No	156 (88.6%)	15 (62.5%)	141 (92.8%)	

* *P* < 0.05.

† Alfred Jaretzki *et al*. 2000.

‡ Muller‐Hemelink *et al*. 1999.

§ Koga *et al*. 1994.

¶ Positive: The serum titer of antiAchR ≥ 0.3 nmol/L. Negative: The serum titer of antiAchR<0.3 nmol/L (Nakajima *et al*. 2008).

†† R0 resection: no residual tumor on microscopy; Non‐R0 resection: microscopic or macroscopic residual tumor.

‡‡ Only patients with MG were included (*N* = 64).

§§ Only patients with lymph node dissection were included (*N* = 41).

¶¶ Only patients with complete resection were included (*N* = 176).

a Student's *t‐*test was used.

b Chi‐square test.

c Linear‐by‐linear association.

d Likelihood ratio detection.

e Fisher's exact test was used.

MG, myasthenia gravis; MGFA, Myasthenia Gravis Foundation of America; anti‐AchR, anti‐acetylcholine receptor; WHO, World Health Organization; MPMT, multiple primary malignant tumors.

### Survival outcomes

**Figure 1:**
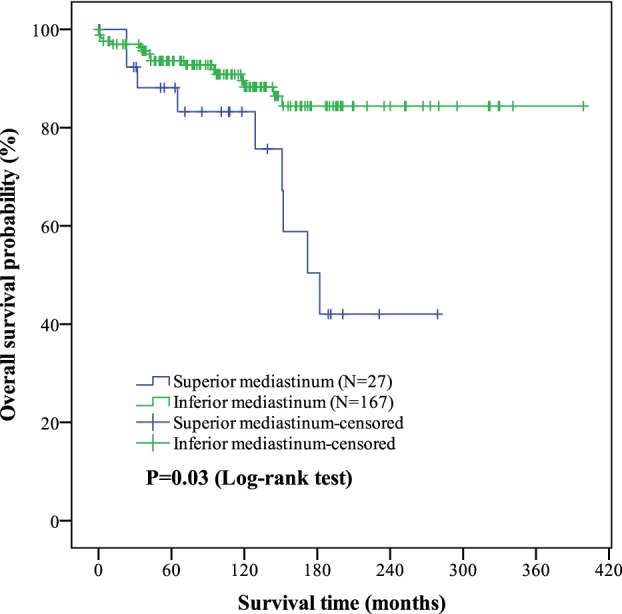
Kaplan‐Meier curves of tumor location (superior/inferior mediastinum) on overall survival (OS) outcomes in thymoma. The mean overall survival in superior and inferior mediastinum of thymoma were (188.5 ± 21.4) months and (349.3 ± 11.7) months. The difference of OS between two groups was significant (*P* = 0.003). Of the 194 patients, the OS was (327.3 ± 13.3) months.

**Figure 2:**
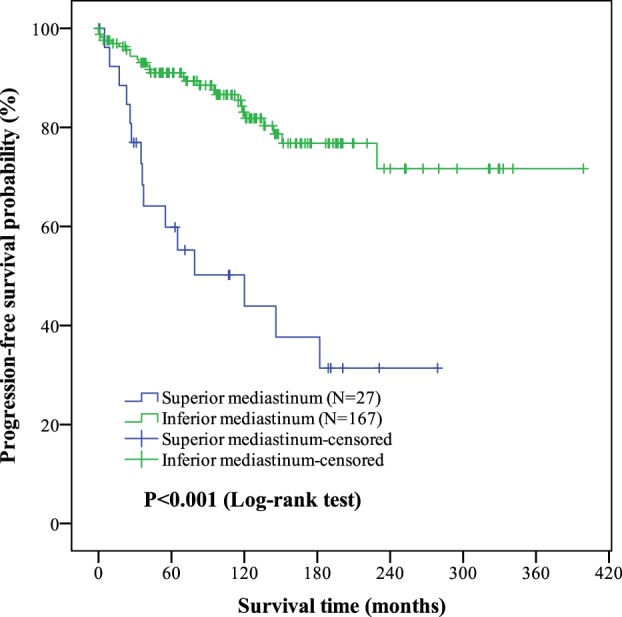
Kaplan‐Meier curves of tumor location (superior/Inferior mediastinum) on progression‐free survival (PFS) outcomes in thymoma. The mean PFS in superior and inferior mediastinum of thymoma were (133.7 ± 23.2) months and (316.2 ± 15.4) months. The differences of PFS between two groups were significant (*P* < 0.001). Of the 194 patients, the PFS was (294.7 ± 14.8) months.

The median follow‐up period was 115.0 months (range: 0–399 months). The OS and PFS were 188.5 ± 21.4 months and 133.7 ± 23.2 months in the superior mediastinum group versus 349.3 ± 11.7 months and 316.2 ± 15.4 months in the inferior mediastinum group, respectively. The differences in OS and PFS between the superior and inferior mediastinum groups were significant (*P* = 0.003 and *P* < 0.001, respectively). Patients with thymomas in the superior mediastinum experienced a significantly worse OS and PFS than those with thymomas in the inferior mediastinum. The Kaplan‐Meier curves are shown in Figures 1 and 2.

**Figure 3:**
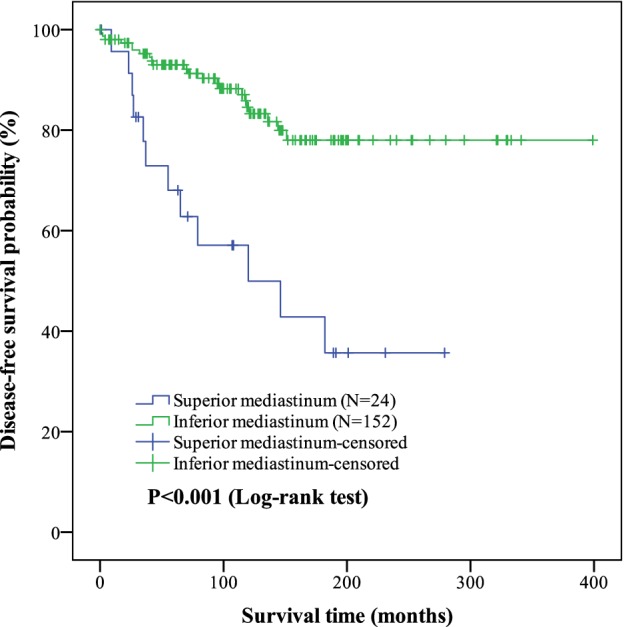
Kaplan‐Meier curves of tumor location (superior/inferior mediastinum) on disease‐free survival (DFS) outcomes in thymoma. The mean DFS in superior and inferior mediastinum of thymoma were (149.3 ± 24.5) months and (330.4 ± 13.5) months. The differences of DFS between two groups were significant (*P* < 0.001). Of the 176 patients, the DFS was (308.1 ± 13.9) months.

DFS was also assessed in this study, but only with patients who underwent an R0 resection. The DFS for patients with superior and inferior mediastinum were 149.3 ± 24.5 days and 330.4 ± 13.5 days, respectively. There was a significant difference in DFS between the two groups (*P* < 0.001). Thymomas in the superior mediastinum were associated with significantly worse DFS. The Kaplan‐Meier curves are shown in Figure 3.

### Prognostic factors for survival outcomes

To verify that the tumor location itself was associated with survival outcomes in patients with thymomas, both univariate and multivariate analysis using a Cox proportional hazard model were performed for OS and PFS.

In the univariate analysis, both OS and PFS were associated with tumor location, age, Masaoka‐Koga stage and preoperative induction therapy (*P* < 0.05). In addition, the tumor diameter, WHO classification and surgical radicality were shown to be predictors of PFS in the univariate analysis (*P* < 0.05). In the multivariate analysis, only tumor location and age were found to be independent prognostic factors of OS (*P* = 0.048 and < 0.001, respectively) and PFS (*P* < 0.001 and 0.002, respectively). Thymomas in the superior mediastinum were associated with a worse OS (HR, 0.371; 95% CI, 0.139–0.989; *P* = 0.048) and PFS (HR, 0.250; 95% CI, 0.117–0.533; *P* < 0.001). Additionally, preoperative induction therapy was shown to be an independent prognostic factor for OS (*P* = 0.030), but not for PFS (*P* = 0.466). The Masaoka‐Koga stage was an independent prognostic factor for PFS (*P* = 0.004) but not for OS (*P* = 0.272). In contrast, sex, MG, anti‐AchR, MPMT, lymph node dissection, and postoperative adjuvant therapy were not prognostic factors in either the univariate or multivariate analysis (*P* > 0.05). The results are presented in Tables 2 and 3.

**Table 2:** Univariate and multivariate analysis of prognostic factors according to OS in thymoma

	OS	Univariate analysis		Multivariate analysis	
Prognostic factors	*n* (%)	HR (95% CI)	*P*‐value	HR (95%CI)	*P*‐value
Tumor location (Superior/Inferior)	18 (66.7)/150 (89.8)	0.318 (0.142–0.715)	0.006*	0.371 (0.139–0.989)	0.048*
Age (continue)	‐	1.070 (1.034–1.107)	<0.001*	1.070 (1.034–1.107)	<0.001*
Tumor diameter (continue)	‐	1.087 (0.953–1.239)	0.215	‐	‐
Sex (Male/Female)	85 (86.7)/83 (86.5)	1.079 (0.499–2.333)	0.847	‐	‐
MG (Yes/No)	54 (84.4)/114 (87.7)	1.136 (0.514–2.510)	0.752	‐	‐
Anti‐AchR (positive/negative)§	53 (85.5)/115 (87.1)	1.008 (0.449–2.263)	0.985	‐	‐
WHO classification(A/AB/B1/B2/B3)†	14 (82.4)/43 (95.6)/57 (89.1)/40 (80.0)/14 (77.8)	1.403 (0.973–2.023)	0.070	1.093 (0.752–1.587)	0.641
Masaoka stage (I/II/III/IV)‡	87 (88.8)/49 (90.7)/18 (75.0)/14 (77.8)	1.541 (1.072–2.214)	0.019 *	1.277 (0.826–1.974)	0.272
MPMT (Yes/No)	21 (77.8)/147 (88.0)	2.218 (0.885–5.556)	0.089	1.894 (0.677–5.297)	0.224
Surgical radicality (R0/non‐R0)¶	153 (86.9)/15 (83.3)	2.179 (0.646–7.353)	0.209	‐	‐
Lymph node dissection (Yes/No)	34 (82.9)/134 (87.6)	1.454 (0.611–3.463)	0.397	‐	‐
Preoperative therapy (Yes/No)	5 (55.6)/163 (88.1)	5.387 (1.823–15.917)	0.002*	3.813 (1.143–12.724)	0.030*
Postoperative therapy (Yes/No)	65 (82.3)/103 (89.6)	1.395 (0.642–3.031)	0.400	‐	‐

* *P* < 0.05.

† Muller‐Hemelink *et al*., 1999.

‡ Koga *et al*., 1994.

§ Positive: The serum titer of Anti‐AchR ≥0.3nmol/L. Negative: The serum titer of Anti‐AchR<0.3nmol/L (Nakajima *et al*. 2008).

¶ R0 resection: no residual tumor on microscopy; Non‐R0 resection: microscopic or macroscopic residual tumor.

OS, overall survival; HR, harzard ratio; CI, confidence interval; MG, myasthenia gravis; anti‐AchR, anti‐acetylcholine receptor; WHO, World Health Organization; MPMT, multiple primary malignant tumors.

**Table 3:** Univariate and multivariate analysis of prognostic factors according to PFS in thymoma

	PFS	Univariate analysis		Multivariate analysis	
Prognostic factors	*n* (%)	HR (95% CI)	*P* value	HR (95%CI)	*P* value
Tumor location (Superior/Inferior)	12 (44.4)/140 (83.8)	0.238 (0.126–0.449)	<0.001*	0.250 (0.117–0.533)	<0.001*
Age (continue)	‐	1.033 (1.009–1.058)	0.007 *	1.043 (1.015–1.071)	0.002*
Tumor diameter (continue)	‐	1.155 (1.061–1.258)	0.001*	1.098 (0.981–1.229)	0.105
Sex (Male/Female)	78 (79.6)/74 (77.1)	1.126 (0.614–2.065)	0.701	‐	‐
MG (Yes/No)	46 (71.9)/106 (81.5)	1.487 (0.806–2.744)	0.204	‐	‐
Anti‐AchR (Positive/Negative)§	45 (72.6)/107 (81.1)	1.395 (0.753–2.585)	0.290	‐	‐
WHO classification (A/ AB/B1/B2/B3)†	13 (76.5)/41(91.1)/51(74.0)/37(74.0)/10(55.6)	1.468 (1.097–1.963)	0.010 *	0.968 (0.710–1.322)	0.840
Masaoka stage (I/II/III/IV)‡	87 (88.8)/46(85.2)/14 (58.3)/5(27.8)	2.452 (1.863–3.227)	<0.001*	1.890 (1.223–2.922)	0.004*
MPMT (Yes/No)	18 (66.7)/134(80.2)	1.953 (0.928–4.111)	0.078	2.105 (0.918–4.829)	0.079
Surgical radicality (R0/non‐R0)¶	142 (80.7)/10(55.6)	4.421 (2.025–9.651)	<0.001*	2.303 (0.791–6.705)	0.126
Lymph node dissection (Yes/No)	28 (68.3)/124(81.0)	1.857 (0.965–3.574)	0.064	0.802 (0.389–1.655)	0.551
Preoperative therapy (Yes/No)	4 (44.4)/148(80.0)	4.122 (1.597–10.639)	0.003*	1.559 (0.473–5.136)	0.466
Postoperative therapy (Yes/No)	59 (74.7)/93(80.9)	1.083 (0.588–1.992)	0.798	‐	‐

* *P* < 0.05.

† Muller‐Hemelink *et al*., 1999.

‡ Koga *et al*., 1994.

§ Positive: The serum titer of anti‐AchR ≥0.3 nmol/L. Negative: The serum titer of anti‐AchR<0.3 nmol/L (Nakajima *et al*. 2008).

¶ R0 resection: no residual tumor on microscopy; Non‐R0 resection: microscopic or macroscopic residual tumor.

PFS, progression‐free survival; HR, harzard ratio; CI, confidence interval; MG, myasthenia gravis; anti‐AchR, anti‐acetylcholine receptor; WHO, World Health Organization; MPMT, multiple primary malignant tumors.

### Risk factors for recurrence

Only the patients with an R0 resection were included in the present analysis of recurrence. For the 176 patients, tumor location, tumor diameter, WHO classification, Masaoka‐Koga stage and preoperative induction therapy were identified as significant variables by the univariate analysis (*P* < 0.05), while tumor location (OR, 0.294; 95% CI, 0.107–0.803; *P* = 0.017) and Masaoka‐Koga stage (OR, 3.355; 95% CI, 1.756–6.409; *P* < 0.001) were the only independent risk factors identified in the multivariate analysis. Recurrence was more likely for thymomas in the superior mediastinum and advanced Masaoka‐Koga stages than for thymomas in the inferior mediastinum (Table 4).

**Table 4:** Univariate and multivariate analysis of risk factors according to recurrence in thymoma

	Recurrence	Univariate analysis		Multivariate analysis	
Prognostic factors	*n* (%)	HR (95% CI)	*P*‐value	HR (95%CI)	*P‐*value
Tumor location (superior/inferior)	9 (37.5)/11(7.2)	0.158 (0.065–0.381)	<0.001*	0.294 (0.107–0.803)	0.017*
Age (continue)	‐	1.002 (0.969–1.035)	0.926	‐	‐
Tumor diameter (continue)	‐	1.236 (1.122–1.361)	<0.001*	1.027 (0.885–1.193)	0.724
Sex (Male/Female)	10 (10.8)/10(12.0)	1.067 (0.444–2.565)	0.884	‐	‐
MG (Yes/No)	10 (18.2)/10(8.3)	2.166 (0.900–5.212)	0.084	1.431(0.521–3.929)	0.486
Anti‐AchR (positive/negative)§	8 (15.4)/12(9.7)	1.536 (0.627–3.760)	0.348	‐	‐
WHO classification (A/AB/B1/B2/B3)†	1 (6.3)/2(4.4)/8(13.8)/4(8.9)/5(41.7)	1.704 (1.110–2.615)	0.015*	0.967 (0.597–1.566)	0.891
Masaoka stage (I/II/III/IV)‡	2 (2.0)/6(11.1)/5(29.4)/7(100.0)	4.131(2.672–6.385)	<0.001*	3.355 (1.756–6.409)	<0.001*
MPMT (Yes/No)	4 (15.4)/16(10.7)	1.655 (0.551–4.971)	0.369	‐	‐
Lymph node dissection (Yes/No)	7 (20.0)/13(9.2)	2.259 (0.901–5.665)	0.082	0.899 (0.334–2.421)	0.833
Preoperative therapy (Yes/No)	5 (55.6)/15(9.0)	10.037 (3.545–28.413)	<0.001*	1.949 (0.510–7.442)	0.329
Postoperative therapy (Yes/No)	11 (15.7)/9(8.5)	1.505 (0.621–3.651)	0.366	‐	‐

* *P* < 0.05.

† Muller‐Hemelink *et al*., 1999.

‡ Koga *et al*., 1994.

§ Positive: The serum titer of anti‐AchR ≥0.3 nmol/L. Negative: The serum titer of anti‐AchR<0.3 nmol/L (Nakajima *et al*. 2008).

HR, harzard ratio; CI, confidence interval; MG, myasthenia gravis; anti‐AchR, anti‐acetylcholine receptor; WHO, World Health Organization; MPMT, multiple primary malignant tumors.

## Discussion

It is particularly important to determine the specific characteristics of thymomas. However, no previous studies have discussed the importance of tumor location in determining clinicopathological features or its relationship to prognosis. While many authors have attempted to identify prognostic factors and risk factors for the recurrence of thymomas, none have identified tumor location as a risk factor.^10^, ^13^, ^29^, ^30^

### Clinicopathological characteristics of thymomas

In the study by Padda *et al*. 33.0% of patients with thymomas had MG, which was similar to the incidence observed in the ITMIG database.^31^ However, thymomas located in the superior mediastinum were more frequently associated with MG than those located in the inferior mediastinum. There is no consensus on the pathophysiological link between thymomas and MG.^32^ However, previous studies have demonstrated a correlation between MG and anti‐AChR antibodies.^33^, ^34^ In our study, there were more patients with positive anti‐AchR in the superior mediastinum group but the difference between the two groups was not statistically significant.

Masaoka‐Koga stage III/IV accounts for approximately 30% of all patients with thymomas.^15^, ^35^, ^36^, ^37^, ^38^ In this study, we found that thymomas located in the superior mediastinum were more likely to be an advanced Masaoka‐Koga stage than those located in the inferior mediastinum. The observation that there were more advanced stages of thymomas in the superior mediastinum can be explained from an anatomical standpoint as follows. Thymomas in the superior mediastinum are closely adjacent to the great vessels, such as the innominate vein, superior vena cava and others, which facilitates the invasion of the thymoma to neighboring organs. Furthermore, the superior mediastinum thymomas surround the great vessels, which makes them rich in blood supply and enables them to become metastatic even early in their disease history.

Progression and recurrence after the initial thymoma is resected is observed in 6.9%–18.0% of patients and can occur even decades after the primary resection.^22^, ^29^, ^39^, ^40^, ^41^, ^42^ In this study, thymomas located in the superior mediastinum were more likely to be associated with disease progression and tumor recurrence than those in the inferior mediastinum. The differences between the two anatomic locations of thymomas can be attributed to the following reasons. The superior mediastinum thymomas are linked to the great vessels which increases the likelihood of hematogenous metastasis when compared to thymomas of the inferior mediastinum. Regarding surgical treatment, the R0 resection is difficult to perform for thymomas in the superior mediastinum, which may also increase the disease progression rate.^38^ Even with an R0 resection of the superior mediastinum thymoma, the probability of an intraoperative tumor implantation is increased, and the progression and recurrence of the patients will be affected accordingly. The clinical stage is often correlated with progression and recurrence rates.^38^, ^43^ Our results from the Masaoka‐Koga stage of the thymomas demonstrated that thymomas located in the superior mediastinum were more likely to be an advanced Masaoka‐Koga stage, which may result in higher progression and recurrence rates for the tumors in the superior mediastinum.

### Survival outcomes of and prognostic factors for thymomas

When compared to tumors located in the inferior mediastinum, the thymomas of the superior mediastinum constituted a small proportion of the thymomas in this case series. However, the patients of the superior mediastinum group had an overall worse prognosis according to our survival analysis, and thus, their thymomas may have been more aggressive in nature. The OS, PFS and DFS in the inferior mediastinum group were largely consistent with previous reports, but those in the superior mediastinum group were worse than what has been previously reported.^14^, ^20^, ^34^ To our knowledge, this is the first study to characterize the relationship between survival outcomes and the tumor location of thymomas.

It is difficult to determine a clear set of prognostic factors for thymomas due to the excellent patient survival. A review of prognostic risk factors for thymomas summarized 29 studies that used multivariate analysis to determine risk factors for survival and showed that the Masaoka‐Koga stage was the most important factor associated with survival outcomes, followed by the WHO classification, surgical radicality and tumor size.^12^In our study, the Masaoka‐Koga stage was regarded as a prognostic factor for PFS and the age for both OS and PFS, which was consistent with previous results.^4^, ^44^, ^45^, ^46^ Additionally, this study is the first demonstration that tumor location can also serve as a prognostic factor for OS and PFS. Thymomas in the superior mediastinum may result in poor survival. This is likely due to thymomas in the superior mediastinum reaching a more advanced Masaoka‐Koga stage, which is associated with an increased disease progression and recurrence, as our results demonstrated. We also found that preoperative induction therapy was associated with a worse prognosis for OS, which may be due to the low number of patients who received this therapy (4.6%) in our study. However, the Japanese Association for Research on the Thymus (JART) reported the same results for preoperative induction therapy. The investigators concluded that this is likely because patients with more advanced staging are more likely to receive preoperative induction treatment.^46^ There were no beneficial effects observed for postoperative adjuvant therapy in OS nor PFS, which is also consistent with previous reports by JART.^46^

### Risk factors for the recurrence of thymomas

The ITMIG established that the tumor recurrence rates are the best measure of treatment efficacy in thymomas from at any site.^47^ Recurrence is highly relevant for thymoma patients and more eligible patients are needed to improve the statistical analysis.

The results from a prospective multicenter database identified associations between higher Masaoka stage and increasing tumor size with increased recurrence rates. Sex, WHO classification, and adjuvant therapy were not risk factors for recurrence.^38^ A review showed that an advanced tumor stage predicted a higher recurrence rate in all the included studies.^12^ However, none of these studies discussed the tumor location as a potential predictive factor for recurrence or disease‐free survival. In this study, the tumor location was categorized as either the superior or inferior mediastinum and the Masaoka‐Koga stage was shown to be an independent risk factor for recurrence in both the univariate and the multivariate analysis. We showed that thymomas in the superior mediastinum were more likely to promote tumor recurrence. The neighboring great vessels and the more advanced Masaoka‐Koga stages of the tumors in the superior mediastinum may contribute to these findings.

Collectively, these results demonstrate that tumor location should be included in the future staging systems for thymomas. Furthermore, there might be differences in the optimal treatment protocols for superior and inferior mediastinum thymomas, such as induction treatments, adjuvant therapies and follow‐up intervals. Future multicenter and prospective studies are necessary to confirm this new proposal.

## Limitations

There were several limitations of this study. First, this was a retrospective study with data that was collected over a period of approximately 40 years, which may have led to different diagnoses from the pathologists and surgeons. Second, the preoperative imaging data generated prior to 1999 could only be collected from chart records, which may be less accurate for distinguishing tumor locations than the information obtained directly from electronic records. Third, we could not include every potential confounding variables in our multivariate model. Additional multicenter prospective studies of the primary tumor location are required to confirm our findings and improve insights into the staging and treatment of thymomas.

In conclusion, this is the first report to demonstrate the significant impacts of tumor location on a range of clinicopathological characteristics and prognosis in patients with thymomas. The clinicopathological features of MG, Masaoka‐Koga stage, disease progression and recurrence were different between the superior and inferior mediastinum thymomas. Importantly, we showed that thymomas in the superior mediastinum were associated with worse survival and more recurrence.

## Disclosure

The authors have no conflicts of interest to disclose.
